# A contextual exploration of healthcare service use in urban slums in Nigeria

**DOI:** 10.1371/journal.pone.0264725

**Published:** 2022-02-25

**Authors:** Olufunke Fayehun, Motunrayo Ajisola, Olalekan Uthman, Oyinlola Oyebode, Abiola Oladejo, Eme Owoaje, Olalekan Taiwo, Oladoyin Odubanjo, Bronwyn Harris, Richard Lilford, Akinyinka Omigbodun, On behalf of the Improving Health in Slums Collaborative

**Affiliations:** 1 Department of Sociology, University of Ibadan, Ibadan, Nigeria; 2 Warwick Medical School, University of Warwick, Warwick, United Kingdom; 3 Department of Community Medicine, University of Ibadan, Ibadan, Nigeria; 4 Department of Geography, University of Ibadan, Ibadan, Nigeria; 5 Nigerian Academy of Science, Yaba, Nigeria; 6 Institute of Applied Health Research, University of Birmingham, Birmingham, United Kingdom; 7 Department of Obstetrics & Gynecology, University of Ibadan, Ibadan, Nigeria; Flinders University, AUSTRALIA

## Abstract

**Introduction:**

Many urban residents in low- and middle-income countries live in unfavorable conditions with few healthcare facilities, calling to question the long-held view of urban advantage in health, healthcare access and utilization. We explore the patterns of healthcare utilization in these deprived neighborhoods by studying three such settlements in Nigeria.

**Methods:**

The study was conducted in three slums in Southwestern Nigeria, categorized as migrant, indigenous or cosmopolitan, based on their characteristics. Using observational data of those who needed healthcare and used in-patient or out-patient services in the 12 months preceding the survey, frequencies, percentages and odds-ratios were used to show the study participants’ environmental and population characteristics, relative to their patterns of healthcare use.

**Results:**

A total of 1,634 residents from the three slums participated, distributed as 763 (migrant), 459 (indigenous) and 412 (cosmopolitan). Residents from the migrant (OR = 0.70, 95%CI: 0.51 to 0.97) and indigenous (OR = 0.65, 95%CI: 0.45 to 0.93) slums were less likely to have used formal healthcare facilities than those from the cosmopolitan slum. Slum residents were more likely to use formal healthcare facilities for maternal and perinatal conditions, and generalized pains, than for communicable (OR = 0.50, 95%CI: 0.34 to 0.72) and non-communicable diseases (OR = 0.61, 95%CI: 0.41 to 0.91). The unemployed had higher odds (OR = 1.45, 95%CI: 1.08 to 1.93) of using formal healthcare facilities than those currently employed.

**Conclusion:**

The cosmopolitan slum, situated in a major financial center and national economic hub, had a higher proportion of formal healthcare facility usage than the migrant and indigenous slums where about half of families were classified as poor. The urban advantage premise and Anderson behavioral model remain a practical explanatory framework, although they may not explain healthcare use in all possible slum types in Africa. A context-within-context approach is important for addressing healthcare utilization challenges in slums in sub-Saharan Africa.

## Introduction

Living conditions, health and lifestyle are associated with place of residence [1–5]. There has been the assumption that urban areas have a health advantage over rural areas for a long time. Many conditions that positively influence health, including health care services, water and sanitation, infrastructure, education and proper housing, are more widely available in urban than rural areas [6–8]. However, the growth of slums in many urban settlements in low and middle-income countries has led to a more substantial proportion of urban residents living in deprived and squalid conditions [5, 8–12], with few formal healthcare facilities, which is at variance with the long-held view of urban advantage in health, healthcare access and health facility utilization. Several studies [7, 10–17] have reported a higher morbidity and mortality burden among residents of urban slums in sub-Saharan Africa (SSA) than non-slum urban and rural areas. With an estimated 62% of the SSA urban population living in slums, exploring healthcare access and utilization among slum residents in SSA becomes critical [18].

There are often many healthcare facilities within cities that provide both formal and informal services. The formal healthcare services are regulated institutions with skilled providers. In contrast, the informal healthcare services are unregistered and poorly regulated, with unskilled providers who claim to have undergone some form of apprenticeship [19]. Some community-based studies in urban slums in Africa and Asia reported that a significant proportion of dwellers received treatment advice and bought drugs from shops, drug sellers and other informal providers. In contrast, very few received treatment from medical doctors [20–22]. Other studies noted limited access to specialized health facilities and preferred healthcare providers in the private sector [23, 24]. One study in a Kenyan setting [25] reported issues of poor or non-compliance by patients and potential patients with formal healthcare treatment programs in the health facility.

There are few studies on healthcare utilization in the slums of lower-middle income countries (LMIC), and what little there is suggest that people get most advice and services from informal providers and private practitioners rather than well-organized public or private services [22, 26–30]. The factors that caused poor health in LMIC also seem to prevent access leading to an inverse care situation. In Nigeria, the formal healthcare delivery system has health facilities categorized into primary, secondary and tertiary levels. For a country of over 200 million people, formal healthcare is delivered in 34, 141 primary health care facilities (HCF), 5,275 secondary (HCF) and 155 tertiary HCF [31]. Furthermore, the financial burden of health care is directly on the population as more than 70% of total health expenditure is out of pocket, while only one in 20 people have health insurance [32].

With about 53 percent of the population living in urban slums in Nigeria [32] and having a substantial burden of mortality and morbidity [33], there is a need to explore likely disparities in healthcare utilization across these deprived neigborhood by studying different slums, which are located in different geographical spaces with different characteristics [34–39]. Direct evidence for such disparities is sparse in Nigeria [34, 35]. We examined the use of formal and informal health care services in three slum sites with different urbanization characteristics in two states in Nigeria. This study is part of a research project undertaken across seven slum sites in four countries in Africa and Asia [38].

## Theoretical framework

The study adopted Andersen’s (1995) behavioral model (ABM) of health service use. The theory is premised on the assumption that various factors determine an individual’s decision to utilize a particular health service provider. These factors [40] include the *environmental* and *population* characteristics of the individual. The environmental characteristics are the type of physical environment in which an individual resides, while the population characteristics refer to social, demographic, and health factors that influence health-seeking behavior. These population factors are further grouped into predisposing, enabling resources and need factors. The predisposing characteristics are the basic features of the study population, such as age, sex and marital status. The enabling factors are conditions that may change based on a personal or social effort; for example, education, employment status, household poverty and coverage by health insurance are enabling factors. The need factor reflects the presenting disease or medical complaints and is usually directly associated with healthcare services [41].

In this study, we explore the interaction of an individual’s environment and population characteristics with health-seeking behavior/outcome, which is the use of formal versus informal healthcare facilities. The environmental characteristic is the type of slum. Population characteristics are predisposing factors (age, gender and marital status); enabling resources- (education, employment status, household poverty measure and health insurance); and the need factor- the presenting medical complaint.

## Materials and methods

This study is part of a multi-country study under the Global Health Research Unit on Improving Health in Slums. The survey methodology and map description for each study site have been published in detail elsewhere [38, 42]. The survey adopted a cross-sectional design within three purposively selected slums in the urbanized cities of Southwestern Nigeria.

### Study setting

We summarized the slum characteristics and reclassified them as follows:

1. Migrant slum/community in Ibadan city of Oyo State, is a resettled community on the edge of the city, built around a major food market. This is a multiethnic population, including many migrants from northern Nigeria. Structures are well-spaced, mostly permanent with variable energy-access, poor sanitation and refuse-filled drains. It has an approximate population of 5,800 people. Most of the 32 health facilities documented in this slum are informal patent medicine vendors (n = 22) and five traditional and spiritual healers. There is one state-run primary healthcare clinic and four small private clinics.
2. Indigenous slum in Ibadan city of Oyo State is centrally located in the historical area of the city, along an old tarred road. Many residents are traders in the three major markets on the site. Mostly permanent but run-down structures, poor sanitation and refuse-filled drains characterize the space. The area is poorly planned with a minimal road network—many health facilities are not easily accessible during emergencies. It has an approximate population of 5,500 people. Most available healthcare is informally provided: patent medicine vendors (n = 15) and traditional and spiritual healers (n = 14) together comprise 80% of the 36 facilities documented on the site. In addition, three state-run primary health clinics (two affiliated to a university teaching hospital) and four small private maternity homes (1–2 beds) operate within the slum.
3. Cosmopolitan slum in Lagos State is a multiethnic population located along the lagoon-front in Lagos, Nigeria’s commercial capital. Residents are mostly educated and employed. Structures are mostly temporary, sanitation and basic services are limited, and the site has a higher crime rate than the others described above. It has an approximate population of 8,100 people. There are three formal healthcare facilities in this slum; one public primary health care centre, one private clinic and one maternity home. Five patent medicine vendors (PMV) and five traditional/spiritual healers also operate in the slum as informal health care providers.

### Participants and sampling

A total of 1500, 1001 and 977 adult participants responded to the survey questions in the migrant, indigenous and cosmopolitan slums, respectively. Of these survey respondents, those who responded that they needed healthcare were 892 (59.7%) in the migrant slum, 573 (52.2%) in the indigenous slum and 497 (50.9%) in the cosmopolitan slum. Out of those who needed care, 880, 560 and 482 participants in the migrant, indigenous and cosmopolitan slum reported receiving care in-patient or out-patient services. For this study, we focused on those who indicated that they needed healthcare, used in-patient and out-patient services and specified the healthcare facility used in the 12 months preceding the survey. Participants relying on home remedies were also excluded from the analysis. Those who met these criteria in the three slums were 763 (migrant), 459 (indigenous) and 412 (cosmopolitan) slum residents.

### Ethical and data collection procedure

Ethical approval was obtained from the Research Ethics Committee of the Oyo State Ministry of Health (AD13/479/657) and of Lagos State (LREC.06/10/993) as well as from the University of Warwick Biomedical and Scientific Research Ethics Sub-Committee (REGO-2017-2043 AM01). In addition, we obtained written consent from each of the participants. The survey instruments were uploaded into an Open Data Kit (ODK) application. All interviews were conducted in either English or the local language spoken in each slum, Yoruba or Hausa. Trained research assistants used the ODK forms to collect information from participants about household healthcare utilization. Field supervisors ensured quality control of data. Data uploaded to the server were cleaned to eliminate errors.

### Dependent and independent variables

#### Dependent variables

The dependent variable for this study is the healthcare facility accessed. Slum residents were asked whether they had sought healthcare in the preceding 12 months and, if so, to provide details about their most recent visit to health services, including where they had sought care. We classified their answers by types of healthcare facility used: formal healthcare facility (public and private hospitals/clinics/primary healthcare centers and registered pharmacies) or informal healthcare facility (patent medicine vendors and traditional care).

#### Independent variables

The independent variables were presenting medical complaints, age, gender, marital status, education, employment status, household poverty and health insurance coverage. Presenting medical complaints were grouped into communicable diseases (including malaria, tuberculosis and HIV), maternal and perinatal conditions, non-communicable diseases (including hypertension, diabetes and cancer) and generalized pain/others. Education was grouped into below secondary; secondary and tertiary. Employment status was categorized into employed and not employed while household poverty was measured as spent less than US$1.90 per day or spent US$1.90 and above per day. The health insurance coverage responses were yes or no). The participant’s age was grouped into young adulthood: 18–35 years; middle age: 36 to 55 years; older adulthood: 56 years or older, Gender of the participant was either male or female and marital status was grouped into married/cohabiting; divorced/separated/widowed; never married/cohabited.

### Statistical analysis

This was an exploratory analysis using observational data of slum residents who indicated that they needed healthcare, used in-patient and out-patient services, and specified the healthcare facility used in the 12 months preceding the survey We used descriptive statistics (frequency and percentages) to show the study participants’ environmental and population characteristics and pattern of health care use among the slum residents. We further explored the odds of formal healthcare service use among different slums by presenting medical complaints, age, sex, marital status, education, employment status and household poverty and health insurance coverage using unadjusted logistic regression. We used IBM SPSS software for the statistical analysis.

## Results

We present the population characteristics of study participants who needed and used health care services in the 12 months preceding the survey in the migrant, indigenous and cosmopolitan slums in Table 1.

**Table 1:** Percentage distribution of population characteristics of participants who needed and sought healthcare in the selected urban slums in Nigeria.

Characteristics	Migrants % (n)	Indigenous % (n)	Cosmopolitan % (n)
*Age*			
Young adulthood: 18–35 years	49.9 (381)	32.5 (149)	43.0 (177)
Middle age: 36–55 years	34.2 (261)	33.8 (155)	39.3 (162)
Older adulthood: 56 year +	15.9 (121)	33.8 (155)	17.7 (63)
*Sex*			
Female	68.3 (521)	54.4 (250)	58.3 (240)
Male	31.7 (242)	45.5 (209)	41.7 (172)
*Marital Status*			
Married/ cohabiting	68.7 (517)	58.1 (262)	65.1 (265)
Divorced/Separated/Widowed	12.4 (93)	30.8 (139)	11.8 (48)
Never married/cohabited	19.0 (143)	11.1 (50)	23.1 (94)
*Education*			
Below Secondary	31.7 (191)	38.4 (138)	14.3 (55)
Secondary and Tertiary	68.3 (411)	61.6 (221)	85.7 (330)
*Employment Status*			
Not employed	28.4 (214)	15.7 (71)	17.9 (73)
Employed	71.6 (539)	84.3 (380)	82.1 (334)
*Household Poverty Measure*			
Less than $1.90 per day	51.6 (394)	49.9 (229)	17.2 (71)
$1.90 and above per day	48.4 (369)	50.1(230)	82.8 (341)
*Health insurance*			
No	96.9 (730)	98.2 (443)	93.4 (380)
Yes	3.1 (23)	1.8 (8)	6.6 (27)

Source: Computed by the authors from data collected in the survey for the study.

The majority of the participants in all the three slums were female, married or cohabiting, educated to at least secondary level and had some form of employment. However, age and income varied among the three slums. The migrant and cosmopolitan slums had more young adults less than 35 years than those in the other age categories. On the contrary, the indigenous slum had more middle-aged and older adults who needed and used healthcare facilities in the 12 months immediately preceding the survey. In addition, household poverty was lower among slum residents in the cosmopolitan slum, where 82.8% of residents spent an aggregate of $1.90 and above per day in their households, compared to 48.4% and 50.1% who were in a similar group in the migrant and indigenous slums, respectively.

**Fig 1:**
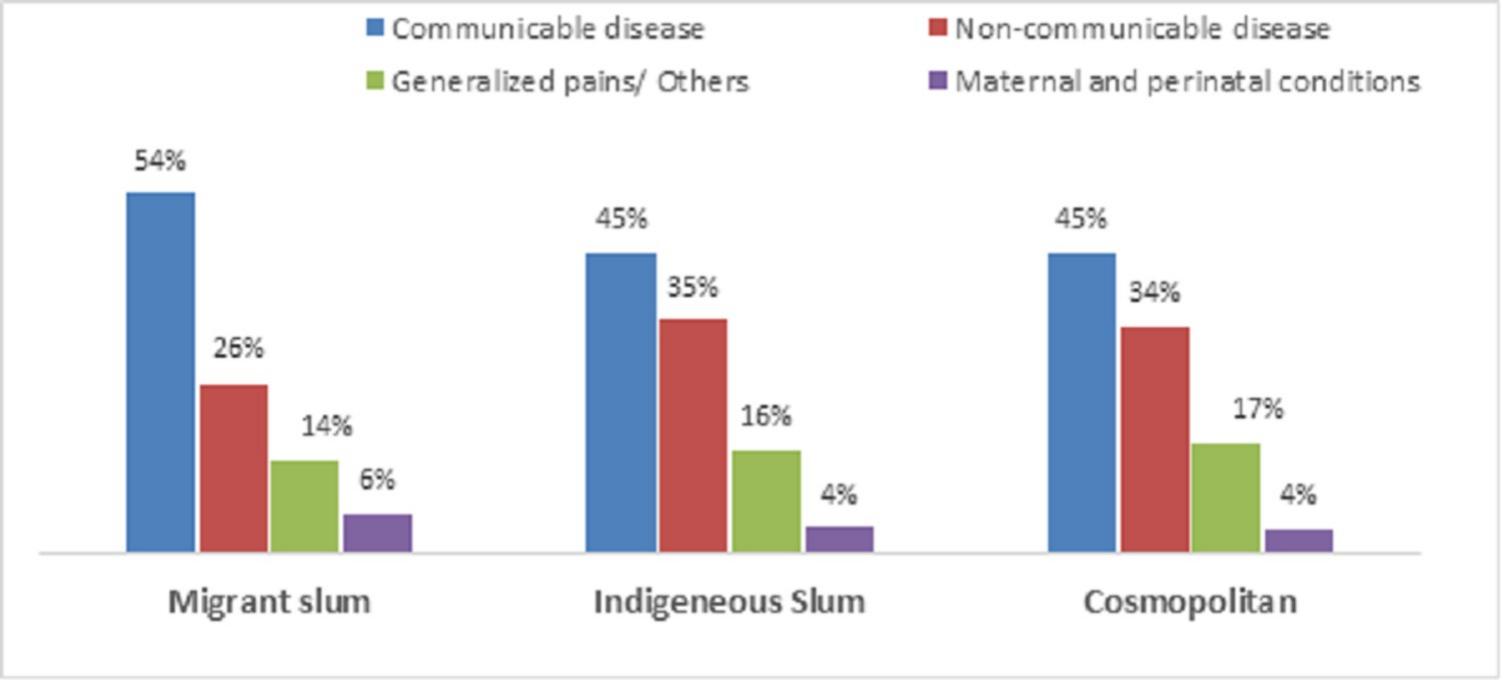
Percentage distribution of presenting medical complaints for in-patient and out-patient services in three urban slums in Nigeria. Source: Drawn by the authors from data collected in the survey for the study.

The presenting medical complaints as a need factor among slum residents were broadly categorized into communicable illnesses, maternal and perinatal conditions, non-communicable disease and generalized pain/others (Fig 1). Communicable diseases were the most common illnesses in all the slums. Maternal and perinatal medical condition-related complaints were the least mentioned in the three slum sites. The migrant slum had the highest proportion of participants that presented with at least one form of communicable disease (54%) and maternal/perinatal condition (6%). The indigenous slum had a higher proportion of non-communicable diseases (NCD) cases (35%) than other slums. The percentage of those whose complaints was generalized pain is higher in the cosmopolitan slum (17%) than the migrant and indigenous slums.

We presented the pattern of the type and ownership of healthcare facilities across the three urban slums in Table 2. Across all the slum sites, the proportion of residents who needed and sought healthcare from the formal health care facilities in the 12 preceding the survey was higher than those who used the informal healthcare facilities. However, a higher proportion of residents in the cosmopolitan slum (83%) used formal healthcare facilities than in the migrant (76.9%) and indigenous (75.8%) slums. Among the formal healthcare facilities in the three slums, the most common healthcare providers used were public sector-owned hospital or healthcare centre, with the highest usage being in the indigenous slum. For the informal health facilities, residents in the three slums patronized patent medicine vendors more than the traditional care centres.

**Table 2:** Percentage distribution of type of healthcare facility used by slum residents on their most recent visit to health services in three urban slums in Nigeria.

	Migrant Slum	Indigenous Slum	Cosmopolitan Slum
**Formal Health Facility**			
Public Hospital/ Clinic/ Health centres	38.4	45.5	34.8
Private Hospital/ Clinic/ Health centres	29.3	19.4	34.8
Pharmacy	9.2	10.9	13.4
*All Formal Health Facilities*	*76*.*9*	*75*.*8*	*83*.*0*
**Informal Health Facility**			
Patent-Medicine Store	20.4	17.4	12.9
Traditional Centres	2.7	6.7	4.1
*All Informal Health Facilities*	*23*.*1*	*24*,*1*	*17*.*0*
Number of slum residents	763	459	412

Source: Computed by the authors from data collected in the survey for the study.

An examination of healthcare facilities broken down by presenting features is given in Table 3. Care for communicable diseases was often sought from formal public and private health facilities or informal patent medicine vendors. The indigenous and the cosmopolitan slums had similar healthcare use patterns when they had NCD, with more residents presenting in public health facilities than others. The migrant slum showed a different pattern, with more NCD complaints seen in formal private health facilities (32.7%) than other health facilities.

**Table 3:** Medical complaints at formal and informal healthcare facilities across the slums.

Health Service Providers	Migrant (%)				Indigenous (%)				Cosmopolitan (%)			
	C	NC	GP	MP	C	NC	GP	MP	C	NC	GP	MP
*Formal HF*												
Public H	39.8	28.8	40.7	59.2	45.7	43.8	48.7	50.0	40.6	33.8	42.5	55.3
Private H	24.3	32.7	38.1	40.8	21.3	14.8	18.4	45.0	25.8	25.7	35.1	40.0
Pharmacy	8.2	14.9	6.2	0.0	6.8	18.2	9.2	0.0	7.8	18.5	7.7	0.0
*Informal HF*												
Patent Medicine Vendors	24.5	22.1	10.6	0.0	20.4	17.6	11.8	0.0	21.8	18.3	8.9	0.0
Traditional	3.2	1.4	4.4	0.0	5.9	5.7	11.8	5.0	4.0	3.6	5.8	4.7
*Number*	*437*	*208*	*113*	*49*	*221*	*176*	*76*	*20*	*845*	*529*	*259*	*85*

**C**-Communicable diseases, **NC**- Non-communicable diseases, **GP**-Generalised pains/others **MP**- Maternal and perinatal conditions

Source: Computed by the authors from data collected in the survey for the study.

Maternal and perinatal conditions, generalized pains and other illnesses were mostly presented at formal health facilities, both public and private, across all the slums. More than three out of four residents who complained of generalized pain used formal health care facilities in the three slums. More than 90% of maternal and perinatal conditions cases used hospitals, clinics or centres.

The unadjusted odds ratio with 95% confidence interval for the logistic regression on formal healthcare facility use in the three urban slums in Nigeria is presented in Table 4.

**Table 4:** Unadjusted odds ratios, with 95% confidence intervals of formal healthcare service use for environmental and population characteristics in urban slums in Nigeria.

Characteristics	Unadjusted Model
*Slum type*	
Migrant	0.70 (0.51–0.97)*
Indigenous	0.65 (0.45–0.93)*
Cosmopolitan (ref.)	1.00
*Presenting medical complaints*	
Communicable disease	0.50 (0.34–0.72)*
Maternal & Perinatal conditions	3.48 (1.21–10.06)*
Non-communicable disease	0.61 (0.41–0.91)*
Generalized pains (ref.)	1.00
*Age*	
Young adulthood: 18–35 years	1.06 (0.79–1.44)
Middle age: 36–55 years	0.92 (0.67–1.25)
Older adulthood: 56 year +(ref.)	1.00
*Sex*	
Female	1.12 (0.89–1.42)
Male (ref.)	1.00
*Marital Status*	
Married/ cohabiting (ref.)	1.00
Divorced/Separated/Widowed	0.85 (0.63–1.15)
Never married/cohabited	0.94 (0.69–1.27)
*Highest level of education*	
Below secondary	1.15 (0.87–1.52)
Secondary and Tertiary (ref.)	1.00
*Employment status*	
Not employed	1.45 (1.08–1.93)*
Employed (ref.)	1.00
*Household poverty measure*	
Less than $1.90 per day	0.79 (0.63–1.00)*
$1.90 and above per day (ref.)	1.00
*Has health insurance*	
No	0.39 (0.17–0.91)*
Yes (ref.)	1.00

OR–Odds ratio. CI–Confidence interval

*p≤0.05 Outcome: ‘formal healthcare facility use:1 versus ‘informal healthcare facility use: 0.

Source: Computed by the authors from data collected in the survey for the study.

Residents from migrant (OR = 0.70, 95% CI 0.51 to 0.97) and indigenous (OR = 0.65, 95% CI 0.45 to 0.93) slums were less likely to have used formal healthcare facilities than those from the cosmopolitan slum. On the presenting medical complaints, all the slum residents were more likely to use formal healthcare facilities for generalized pain/other complaints, and for maternal and perinatal conditions than for communicable diseases (OR = 0.50, 95% CI 0.34 to 0.72) and non-communicable diseases (OR = 0.61, 95% CI 0.41 to 0.91). The differences in the odds of using formal health care facility by the predisposing factors, age, gender and marital status were not so distinct in this study. But enabling resources such as employment and health insurance coverage showed different odds of using formal health care facilities. Slum residents who were not employed had higher odds of using formal healthcare facilities than those currently employed at the time of the survey (OR = 1.45, 95% CI 1.08 to 1.93). Also, those with health insurance coverage had a higher likelihood of using formal healthcare services than those with no health insurance (OR = 0.39, 95% CI 0.17 to 0.91).

## Discussion

The findings from this study on the use of formal and informal health care services when seeking care for medical complaints in three different urban slums in Nigeria affirmed the postulations in Andersen’s Behavior Model theory that environment and population factors are important in an individual’s decision to utilize a particular health service provider. The type of slum as an environmental factor in this study showed distinct differences in formal health care services in migrant, indigenous and cosmopolitan slums in Nigeria. The cosmopolitan slum, situated in a major financial center and economic hub of the country, had a higher proportion of formal health care facility usage than the other slums which are in a different city in another state. In addition to the environmental factor, the population enabling resources such as household wealth and access to health insurance are more pronounced in the cosmopolitan slum where formal healthcare utilization was most prevalent. This broadly suggests that the “urban advantage thesis” remains a viable explanatory framework, although it may not explain healthcare use in all possible slum types in Africa [43–45].

Just as a previous study [27] in a slum population had reported that one of the determinants for accessing reproductive health care was the availability of resources at the household level, relative household poverty among these urban slum residents was predictive of whether they would use public health facilities rather than private ones. Participants living in poverty (household spending of less than $1.90 per day) [46] were less likely to use formal healthcare facilities. The other barrier to the affordability of healthcare services for many slum residents is that most of them are without health insurance. Those with health insurance use formal healthcare services more than those without. However, there is a paradox in the fact that the unemployed had higher odds of seeking care in the formal sector despite relatively lower household wealth. This suggests some nuances in health-seeking behavior of the slum residents, with a tendency to seek cheaper formal health care options in government health facilities such as subsidized consultation and medication fees, rather than in privately owned health facilities, as seen in another study in Nigeria [47] where most out-of-pocket spending for health care were found among those with informal unemployment who visit public healthcare facilities.

Population characteristics such as age, sex, marital status were not significant predictors of formal healthcare facility use in the three types of urban slums examined. This contradicts findings from previous studies [48–50] on utilization of healthcare in African and Asian slums where age, sex and marital status were significantly associated with the use of health facilities. These differences in findings may be as a result of the study population, as well as other determinants of health service utilization [51].

The presenting medical complaints at the survey time were also a significant predictor of formal health care utilization across all the slums. The most typical illnesses across all the slums were communicable diseases such as malaria, tuberculosis and other infections. Communicable diseases were often presented in a combination of formal (public and private), and few informal (patent medicine vendors) health facilities across all the three slums. Other studies [8, 52] in low and middle-income countries have shown similar observations among those presenting with communicable diseases and their use of healthcare facilities [8, 52–54]. For example, a recent slum study in India [55] observed significant differences in utilization of formal (private and public) health facilities in treating chronic illnesses, which raises a question of perceived severity of illness by the residents and health care providers.

Public hospitals, clinics or centres were the most visited health facilities for medical complaints relating to non-communicable diseases, maternal and perinatal conditions, and generalized pains in all the slums. Given that generalized pain is challenging to diagnose, especially when it persists, people recognize a need to consult a formal provider. On the other hand, if people held health beliefs about specific symptoms associated with common communicable diseases, whether medically accurate or not, they may perhaps initially try to address these informally. Although the results align with previous findings [56] on slum residents using a combination of private and public health facilities for most of their health conditions, it has implications for referral systems between informal and formal providers in the slum sites. In the informal sector, PMVs are often the first point of community contact, including in our study sites where a higher proportion of visits to pharmacies/PMVs was for new conditions than existing conditions [33]. This finding, as also suggested in our previous study [57], has health system implications, which might be to seek a way of including PMVs in the referral system, with provision of training opportunities for them and implementation of quality control measures.

For maternal and perinatal conditions, slum residents have higher odds of using formal healthcare facilities for maternal and perinatal conditions than other medical conditions. Perhaps, this is because formal healthcare services are often regulated institutions with skilled healthcare providers for maternal and child health, or it may be due to the longer-term investments made by governments on maternal and child health programs [19, 56, 58, 59]. These include free services, community awareness/mobilization and health promotion campaigns directed at maternal child health issues in the country.

In our previous study [57] on selected slum sites in Africa and Asia, we found that visits to pharmacies (formal and informal-PMVs) were less frequent than visits to doctors or nurses in the three Nigerian sites, as well one of two Kenyan slums and a slum in Pakistan. Also, for this study, formal health care facilities utilization was high in urban slums of Nigeria; more than seven out of ten slum residents used these formal health facilities, whether public or private, when having medical complaints. While informal health facilities were least patronized, patent medicine vendors were visited more in migrant and indigenous slums than among cosmopolitan slum residents. For this study, the slum locations’ characteristics seem to shape utilization, knowledge that can facilitate policy changes to take advantage of population preferences in improving health care accessibility.

In Nigeria, malaria is a very common communicable disease whose common symptoms are often recognized by those affected and many often seek care from PMVs before going to formal health facilities when symptoms persist. Hypertension is the leading non-communicable disease in Nigeria, affecting nearly three in ten adults, with slightly higher prevalence in urban areas [60]. Many patients with hypertension simply obtain their usual medication from medicine stores without visiting their formal healthcare provider. One limitation of this study is that neither malaria nor hypertension were isolated as a separate value. We cannot categorically state what proportion of communicable diseases were presented as malaria or what proportion of people with non-communicable disease were hypertensive in the three slums sites. Another limitation of this study is that while the sample size for the entire survey was large enough to support quite precise summaries of different characteristics in the population, the same does not necessarily apply to the sub-group described here. The results presented are therefore exploratory, based on observational data. Further studies are needed to define more precisely the attributes in these slum residents that predict their approaches to health service utilization. There is also a need for further examination of the informal healthcare services and their use in the slums, which residents might have considered as part of their everyday health maintenance practices and not related to incidents of ill health or medical complaints.

## Conclusion

The characteristics of the slum is important in explaining the extent of the use of formal healthcare services in Nigeria. The cosmopolitan slum situated in a major financial center and economic hub of the country had a higher proportion of formal healthcare facility usage than migrant and indigenous slums where about half of families were classified as poor. The urban advantage and Anderson behavioral model premises are practical explanatory frameworks, although they may not explain healthcare use in all possible slum types in Africa. A context-within-context approach is important for addressing the healthcare utilization challenges in slums in sub-Saharan Africa.

## References

[1] Montgomery MR, Hewett PC. Urban poverty and health in developing countries: Household and neighborhood effects. Demography. 2005 Aug;42(3):397–425. doi: 10.1353/dem.2005.0020 16235606

[2] Montgomery MR, Stren R, Cohen B, Reed HE. Cities transformed: demographic change and its implications in the developing world. Routledge; 2013 Oct 31.

[3] Sampson RJ, Morenoff JD, Gannon-Rowley T. Assessing “neighborhood effects”: Social processes and new directions in research. Annual Review of Sociology. 2002 Aug;28(1):443–78.

[4] Kessides C. The urban transition in Sub-Saharan Africa: Implications for economic growth and poverty reduction. Washington, DC: Cities Alliance; 2006 Aug 30.

[5] Martinez J, Mboup G, Sliuzas R, Stein A. Trends in urban and slum indicators across developing world cities, 1990–2003. Habitat International. 2008 Mar 1;32(1):86–108.

[6] Bocquier P, Madise NJ, Zulu EM. Is there an urban advantage in child survival in sub-Saharan Africa? Evidence from 18 countries in the 1990s. Demography. 2011 May 1;48(2):531–58. doi: 10.1007/s13524-011-0019-2 21590463

[7] Kimani-Murage EW, Ngindu AM. Quality of water the slum dwellers use: the case of a Kenyan slum. Journal of Urban Health. 2007 Nov 1;84(6):829–38. doi: 10.1007/s11524-007-9199-x 17551841 PMC2134844

[8] Ezeh A, Oyebode O, Satterthwaite D, Chen YF, Ndugwa R, Sartori J, et al. The history, geography, and sociology of slums and the health problems of people who live in slums. The Lancet. 2017 Feb 4;389(10068):547–58. 10.1016/S0140-6736(16)31650-6 27760703

[9] Sverdlik A. Ill-health and poverty: a literature review on health in informal settlements. Environment and urbanization. 2011 Apr;23(1):123–55.

[10] Fink G, Günther I, Hill K. Slum residence and child health in developing countries. Demography. 2014 Aug 1;51(4):1175–97. doi: 10.1007/s13524-014-0302-0 24895049

[11] Rice J, Rice JS. The concentration of disadvantage and the rise of an urban penalty: urban slum prevalence and the social production of health inequalities in the developing countries. International Journal of Health Services. 2009 Oct;39(4):749–70. doi: 10.2190/HS.39.4.i 19927413

[12] Günther I, Harttgen K. Deadly cities? Spatial inequalities in mortality in sub‐Saharan Africa. Population and Development Review. 2012 Sep;38(3):469–86.

[13] Mberu BU, Haregu TN, Kyobutungi C, Ezeh AC. Health and health-related indicators in slum, rural, and urban communities: a comparative analysis. Global health action. 2016 Dec 1;9(1):33163. 27924741 10.3402/gha.v9.33163 PMC5141369

[14] Fotso JC. Urban–rural differentials in child malnutrition: trends and socioeconomic correlates in sub-Saharan Africa. Health & place. 2007 Mar 1;13(1):205–23. doi: 10.1016/j.healthplace.2006.01.004 16563851

[15] Abuya BA, Ciera J, Kimani-Murage E. Effect of mother’s education on child’s nutritional status in the slums of Nairobi. BMC pediatrics. 2012 Dec;12(1):1–10. doi: 10.1186/1471-2431-12-80 22721431 PMC3444953

[16] Taffa N, Chepngeno G. Determinants of health care seeking for childhood illnesses in Nairobi slums. Tropical Medicine & International Health. 2005 Mar;10(3):240–5. doi: 10.1111/j.1365-3156.2004.01381.x 15730508

[17] Penrose K, Castro MC, Werema J, Ryan ET. Informal urban settlements and cholera risk in Dar es Salaam, Tanzania. PLoS Neglected Tropical Diseases. 2010 Mar 16;4(3):e631. doi: 10.1371/journal.pntd.0000631 20300569 PMC2838775

[18] Amegah AK. Slum decay in Sub-Saharan Africa: Context, environmental pollution challenges, and impact on dweller’s health. Environmental Epidemiology. 2021 Jun;5(3). 10.1097/EE9.0000000000000158 PMC8196122 34131619

[19] Sudhinaraset M, Ingram M, Lofthouse HK, Montagu D. What is the role of informal healthcare providers in developing countries? A systematic review. PloS One. 2013 Feb 6;8(2):e54978. doi: 10.1371/journal.pone.0054978 23405101 PMC3566158

[20] Liu Y, Zhong L, Yuan S, van de Klundert J. Why patients prefer high-level healthcare facilities: a qualitative study using focus groups in rural and urban China. BMJ global health. 2018 Sep 1;3(5):e000854. doi: 10.1136/bmjgh-2018-000854 30258653 PMC6150133

[21] Manna B, Nasrin D, Kanungo S, Roy S, Ramamurthy T, Kotloff KL, et al. Determinants of health care seeking for diarrheal illness in young children in urban slums of Kolkata, India. The American Journal of Tropical Medicine and Hygiene. 2013 Jul 10;89(1 Suppl):56. doi: 10.4269/ajtmh.12-0756 23629936 PMC3748502

[22] Siddiqi KA, Dana T, Hasan K, Haider MR. Mother’s Healthcare Response to Child Illness: A Slum-Based Cross-Sectional Study in Dhaka City, Bangladesh. The Columbia University Journal of Global Health. 2017 Dec 21;7(2):16–25.

[23] Memon ZA, Pach A, Rifkin M, Han OP, Stanton B, Clemens J, et al. Health care preferences for children with typhoid fever in two slum communities in Karachi, Pakistan. Southeast Asian J Trop Med Public Health. 2008 Nov 1;39(6):1110–25. 19062704

[24] Hasan IJ, Khanum A. Health care utilization during terminal child illness in squatter settlements of Karachi. Journal of Pakistan Medical Association. 2000;50(12):405. 11191439

[25] Werner ME, van de Vijver S, Adhiambo M, Egondi T, Oti SO, Kyobutungi C. Results of a hypertension and diabetes treatment program in the slums of Nairobi: a retrospective cohort study. BMC health services research. 2015 Jun;15(1):1–9. doi: 10.1186/s12913-015-1167-7 26577953 PMC4650397

[26] Seth T, Kotwal A, Thakur R, Singh P, Kochupillai V. Common cancers in India: knowledge, attitudes and behaviours of urban slum dwellers in New Delhi. Public Health. 2005 Feb 1;119(2):87–96. doi: 10.1016/j.puhe.2004.05.013 15694955

[27] Bhanderi MN, Kannan S. Untreated reproductive morbidities among ever married women of slums of Rajkot City, Gujarat: the role of class, distance, provider attitudes, and perceived quality of care. Journal of Urban Health. 2010 Mar 1;87(2):254–63. doi: 10.1007/s11524-009-9423-y 20108049 PMC2845825

[28] Nahar S, Banu M, Nasreen HE. Women-focused development intervention reduces delays in accessing emergency obstetric care in urban slums in Bangladesh: a cross-sectional study. BMC pregnancy and childbirth. 2011 Dec;11(1):1–0. doi: 10.1186/1471-2393-11-11 21276263 PMC3045375

[29] Adane M, Mengistie B, Mulat W, Kloos H, Medhin G. Utilization of health facilities and predictors of health-seeking behavior for under-five children with acute diarrhea in slums of Addis Ababa, Ethiopia: a community-based cross-sectional study. Journal of Health, Population and Nutrition. 2017 Dec;36(1):1–2. 10.1186/s41043-017-0085-1 PMC5381138 28376916

[30] Černauskas V, Angeli F, Jaiswal AK, Pavlova M. Underlying determinants of health provider choice in urban slums: results from a discrete choice experiment in Ahmedabad, India. BMC health services research. 2018 Dec;18(1):1–1. doi: 10.1186/s12913-017-2770-6 29921260 PMC6006661

[31] Federal Ministry of Health. Nigeria Health Facility Registry (HFR) [Internet]. 2019 [cited 2021 Oct 4]. Available from: https://hfr.health.gov.ng/statistics/tables

[32] United Nations Human Settlements Programme (UN-HABITAT). Population living in slums (% of urban population)–Nigeria [Internet]. 2021 [cited 2021 Oct 4]. Available from: https://data.worldbank.org/indicator/EN.POP.SLUM.UR.ZS?end=2018&locations=NG&start=2006

[33] Ahmed S.A.S., Ajisola M., Azeem K., Bakibinga P., Chen Y.F., Choudhury N.N., et al., 2020. Impact of the societal response to COVID-19 on access to healthcare for non-COVID-19 health issues in slum communities of Bangladesh, Kenya, Nigeria and Pakistan: results of pre-COVID and COVID-19 lockdown stakeholder engagements. *BMJ Global Health*, 5(8), p.e003042. doi: 10.1136/bmjgh-2020-003042 32819917 PMC7443197

[34] Oladipo JA. Utilization of health care services in rural and urban areas: a determinant factor in planning and managing health care delivery systems. African health sciences. 2014 Jun 11;14(2):322–33. doi: 10.4314/ahs.v14i2.6 25320580 PMC4196405

[35] Onyeonoro UU, Ogah OS, Ukegbu AU, Chukwuonye II, Madukwe OO, Moses AO. Urban–rural differences in health-care-seeking pattern of residents of Abia state, Nigeria, and the implication in the control of NCDS. Health services insights. 2016 Jan;9:HSI-S31865. 10.4137/HSI.S31865 PMC5053202 27721654

[36] World Health Organization. World health statistics 2019: monitoring health for the SDGs, sustainable development goals. World Health Organization; 2019.

[37] National Population Commission (NPC) [Nigeria] and ICF. *Nigeria Demographic and Health Survey 2018 Key Indicators Report*. Abuja, Nigeria, and Rockville, Maryland, USA: NPC and ICF. 2019

[38] Improving Health in Slums Collaborative. A protocol for a multi-site, spatially-referenced household survey in slum settings: methods for access, sampling frame construction, sampling, and field data collection. *BMC Medical Research Methodology* 2019 (19**)** 109. 10.1186/s12874-019-0732-x 31146676 PMC6543601

[39] Habitat UN. State of the world’s cities 2012/2013: Prosperity of cities. Routledge; 2013 Oct 23.

[40] Andersen RM. Revisiting the behavioral model and access to medical care: does it matter?. Journal of Health and Social Behavior. 1995 Mar 1:1–0. 7738325

[41] Van Doorslaer E, Wagstaff A, Van der Burg H, Christiansen T, De Graeve D, Duchesne I, et al. Equity in the delivery of health care in Europe and the US. Journal of health economics. 2000 Sep 1;19(5):553–83. doi: 10.1016/s0167-6296(00)00050-3 11184794

[42] Yeboah G, Porto de Albuquerque J, Troilo R, Tregonning G, Perera S, Ahmed SA, et al. Analysis of Open Street Map Data Quality at Different Stages of a Participatory Mapping Process: Evidence from Slums in Africa and Asia. ISPRS International Journal of Geo-Information. 2021 Apr;10(4):265.

[43] Okedo-Alex IN, Akamike IC, Ezeanosike OB, Uneke CJ. Determinants of antenatal care utilisation in sub-Saharan Africa: a systematic review. BMJ open. 2019 Oct 1;9(10):e031890. doi: 10.1136/bmjopen-2019-031890 31594900 PMC6797296

[44] Fagbamigbe AF, Idemudia ES. Barriers to antenatal care use in Nigeria: evidences from non-users and implications for maternal health programming. BMC pregnancy and childbirth. 2015 Dec;15(1):1–10. doi: 10.1186/s12884-015-0527-y 25885481 PMC4407543

[45] Dimbuene ZT, Amo-Adjei J, Amugsi D, Mumah J, Izugbara CO, Beguy D. Women’s education and utilization of maternal health services in Africa: a multi-country and socioeconomic status analysis. Journal of biosocial science. 2018 Nov;50(6):725–48. doi: 10.1017/S0021932017000505 29103388

[46] Atkinson AB. Monitoring global poverty: Report of the commission on global poverty. World Bank, Washington. 2017.

[47] Aregbeshola BS, Khan SM. Out-of-pocket health-care spending and its determinants among households in Nigeria: a national study. Journal of Public Health. 2021 Aug;29(4):931–42.

[48] Owusu-Ansah FE, Tagbor H, Togbe MA. Access to health in city slum dwellers: The case of Sodom and Gomorrah in Accra, Ghana. African Journal of Primary Health Care and Family Medicine. 2016 Jan 1;8(1):1–7. doi: 10.4102/phcfm.v8i1.822 27247151 PMC4827165

[49] Amiresmaili M, Yazdi-Feyzabadi V, Heidarijamebozorgi M. Health services utilization among slum dwellers: An experience from Iran. Journal of Education and Health Promotion. 2019;8: 210. doi: 10.4103/jehp.jehp_358_19 31807600 PMC6852376

[50] Aleemi AR, Khaliqui H, Faisal A. Challenges and patterns of seeking primary health care in slums of Karachi: a disaster lurking in urban shadows. Asia Pacific Journal of Public Health. 2018 Jul;30(5):479–90. doi: 10.1177/1010539518772132 29717899

[51] Rout SK, Sahu KS, Mahapatra S. Utilization of health care services in public and private healthcare in India: causes and determinants. International Journal of Healthcare Management. 2021 Apr 3;14(2):509–16.

[52] Obuku EA, Meynell C, Kiboss-Kyeyune J, Blankley S, Atuhairwe C, Nabankema E, et al. Socio-demographic determinants and prevalence of Tuberculosis knowledge in three slum populations of Uganda. BMC Public Health. 2012 Dec;12(1):1–9. doi: 10.1186/1471-2458-12-536 22824498 PMC3507884

[53] Sur D, von Seidlein L, Manna B, Dutta S, Deb AK, Sarkar BL, et al. The malaria and typhoid fever burden in the slums of Kolkata, India: data from a prospective community-based study. Transactions of the Royal Society of Tropical Medicine and Hygiene. 2006 Aug 1;100(8):725–33. doi: 10.1016/j.trstmh.2005.10.019 16455118

[54] Akinwale OP, Oyefara LJ, Adejoh P, Adeneye AA, Adeneye AK, Musa ZA, et al. Survey of hypertension, diabetes and obesity in three Nigerian urban slums. Iranian journal of public health. 2013 Sep;42(9):972. 26060658 PMC4453893

[55] Adams AM, Islam R, Yusuf SS, Panasci A, Crowell N. Healthcare seeking for chronic illness among adult slum dwellers in Bangladesh: A descriptive cross-sectional study in two urban settings. PloS one. 2020 Jun 15;15(6):e0233635. doi: 10.1371/journal.pone.0233635 32542043 PMC7295220

[56] Naydenova E, Raghu A, Ernst J, Sahariah SA, Gandhi M, Murphy G. Healthcare choices in Mumbai slums: A cross-sectional study. Wellcome Open Research. 2017;2. doi: 10.12688/wellcomeopenres.13127.2 30027122 PMC6039940

[57] Improving Health in Slums Collaborative., Watson S. Pharmacies in informal settlements: a retrospective, cross-sectional household and health facility survey in four countries. *BMC Health Serv Res* 2021 21(1):1–10. doi: 10.1186/s12913-021-06937-9 34503501 PMC8431901

[58] Karim M, Farah S. Maternal Health Care Practices among Mothers of a Selected Slum in Dhaka City. Journal of Enam Medical College. 2015 Nov 10;5(3):166–9.

[59] Onwujekwe O, Obi F, Ichoku H, Ezumah N, Okeke C, Ezenwaka U, et al. Assessment of a free maternal and child health program and the prospects for program re-activation and scale up using a new health fund in Nigeria. Niger J Clin Pract. 2019 Nov 1;22(11):1516–29. doi: 10.4103/njcp.njcp_503_18 31719273

[60] Adeloye D, Basquill C, Aderemi AV, Thompson JY, Obi FA. An estimate of the prevalence of hypertension in Nigeria: a systematic review and meta-analysis. Journal of hypertension. 2015 Feb 1;33(2):230–42. doi: 10.1097/HJH.0000000000000413 25380154

